# Evaluation of eye and serum findings in different waters in rabbits by drowning and submersion modeling

**DOI:** 10.55730/1300-0144.5764

**Published:** 2023-11-11

**Authors:** Aziz YILMAZ, Erdem HÖSÜKLER, Abdulgani KAYMAZ, Ahmet Yücel ÜÇGÜL, Zehra Zerrin ERKOL

**Affiliations:** 1 Department of Forensic Medicine, Bolu Abant İzzet Baysal University İzzet Baysal Training and Research Hospital, Bolu, Turkiye; 2 Department of Forensic Medicine, Faculty of Medicine, Bolu Abant İzzet Baysal University, Bolu, Turkiye; 3 Department of Eye Diseases, Kırşehir Training and Research Hospital, Kırşehir, Turkiye; 4 Department of Eye Diseases, Faculty of Medicine, Bolu Abant İzzet Baysal University, Bolu, Turkiye

**Keywords:** Salt water, fresh water, drowning, immersion, postmortem vitreus humour, sodium, chloride, magnesium

## Abstract

**Background/aim:**

This study investigated serum, vitreous, and anterior chamber fluid electrolyte changes, corneal thickness (CT), corneal volume (CV), anterior chamber volume (ACV), and anterior chamber depth (ACD) as an auxiliary diagnostic method in the identification of drowning in fresh or salt water.

**Materials and methods:**

The study used 35 healthy, adult, male, white New Zealand rabbits, seperated into five groups (control, saltwater drowning (SWD), saltwater immersion (SWI), freshwater drowning (FWD), freshwater immersion (FWI)). CT, CV, ACV, and ACD measurements were made with Pentacam topography at 0, 2, and 4 h in all groups. Magnesium (mg), sodium (Na), and chlorine (Cl) were measured in the blood at 0 and 2 h, and in blood, vitreous fluid, and humor aqueous at 4 h.

**Results:**

It was determined that CT, CV, ACV, and ACD are not of great value in drowning diagnosis and are affected by the fresh or salt water rather than drowning. Vitreous Na, Cl, and Mg levels are ineffective in determining drowning after one h. Anterior chamber fluid may provide valuable information in the differentiation freshwater – saltwater drownings at the 4th h in corpses retrieved from water.

**Conclusion:**

Anterior chamber fluid Na and Cl levels, especially in corpses removed from salt water, can be an easily used test that can help diagnose drowning.

## 1. Introduction

Drowning is a serious hazard to public health and one of the main causes of injury-related deaths [1]. Low- and middle-income countries are the most affected by unintentional drowning deaths, and it is estimated that 91% of unintentional drownings occur in these countries [2]. While the annual death rate due to drowning in Türkiye was 0.04 per hundred thousand in 2017, it increased to 0.08 per hundred thousand in 2019 [1]. Of all asphyxia deaths, drowning is the condition most frequently needed to confirm or exclude specific causes of death [3]. The cause of death of a corpse removed from water may be drowning, trauma, drugs, natural disease, or other causes. Only after these other causes have been excluded can a drowning diagnosis be made [4]. Although drowning is one of the common causes of death in forensic practice, corpses removed from the water are difficult to diagnose due to nonspecific findings at autopsy, and a thorough autopsy and detailed investigation of the crime scene are required. In addition, despite previous studies, there is no well-proven diagnostic test for drowning [5]. Therefore, although many diagnostic methods have been analyzed, postmortem diagnosis of drowning remains one of the most difficult areas in current forensic pathology [3]. The reason for the use of chemical markers as complementary research in the identification of the cause of death in drowning is based on the analysis of the elements in the liquid that pass from the denser environment to the living organism and the analysis of hemodilution [6]. Therefore, electrolyte changes in blood, pericardial fluid, pleural effusion, sphenoid sinus fluid, and vitreous fluid have been evaluated [7–11]. In addition, pulmonary surfactant, lung weight, diatom tests, postmortem tomography, and aquaporins have also been suggested as complementary tests in the identification of drowning [11–14]. This study’s objective was to assess serum, vitreous and anterior chamber fluid electrolyte changes, corneal thickness (CT), corneal volume (CV), anterior chamber volume (ACV), and anterior chamber depth (ACD) as an auxiliary diagnostic method in the identification of drowning in fresh or salt water.

## 2. Materials and methods

This study was carried out in Bolu Abant İzzet Baysal University Experimental Animals Research and Application Centre. Ethics committee approval was obtained for the study from Bolu Abant İzzet Baysal University Animal Research Local Ethics Committee dated 05.05.2021 and numbered 2021/16. While determining the number of cases, a simulation was made for sample size calculation. It was determined that the minimum number of subjects required to find a significant difference under the best conditions was seven experimental units (rabbits) for each group (p = 0.022). The study used 35 healthy, adult, male, white New Zealand rabbits, each weighing approximately 3–5 kg, obtained from Bolu Abant İzzet Baysal University Experimental Animals Research and Application Centre were used. Before starting the study, it was confirmed that the cornea and other eye findings of the rabbits to be used in the experiment were healthy. The water samples used in the study were 50 mL of sea water for salt water and 50 mL of lake water for fresh water.

**Figure 1:**
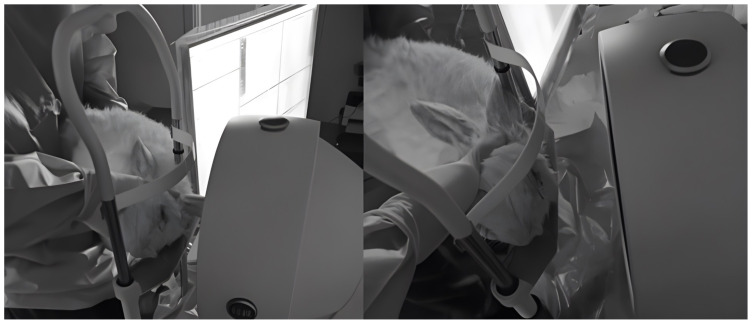
Measurement of eye values of rabbits with pentacam topography.

The animals were separated into five groups (control, saltwater drowning, saltwater immersion, freshwater drowning, freshwater immersion). All of the experimental animals were anesthetized with 35/5 mg/kg ketamine/xylazine. The animals in the control, freshwater immersion, and saltwater immersion groups were sacrified by the administration of carbon dioxide after anesthesia. Then, the subjects in the fresh and salt water immersion group were immersed in the water. Subjects in the drowning groups were held by ears under fresh and saltwater under anesthesia until respiratory depression. Death was confirmed by taking ECG using a power lab device, followed by observation of respiratory parameters. Subjects in all groups except the control group were kept underwater for 4 h. CT, CV, ACV, and ACD measurements were made with pentacam topography at 0, 2, and 4 h in all groups (Figure 1). In addition, 1.5 mL of blood was taken at 0, 2, and 4 h in all the groups (from the ear at 0 h, by intracardiac puncture at 2 and 4 h). At the postmortem 4th h, 1 mL of vitreous fluid and anterior chamber fluid was taken. All samples taken were kept in the freezer at −80 °C until analyses were conducted. Magnesium (Mg) was measured from serum, anterior chamber fluid and vitreous fluid using the colorimetric method on an Otto Scientific OttoBC151 device. Sodium (Na) and Chlorine (Cl) were measured on a Siemens device using the Ion Selective Electrode method.

### 2.1. Statistical methods

Statistical analysis was performed using Statistical Package For Social Science (SPSS), version 21 software (IBM SPSS Statistics for Windows, Version 20.0, Armonk, NY, USA). The conformity of the variables to normal distribution was examined using visual (histogram plots, boxplots) and analytical methods (Kolmogorov-Smirnov/Shapiro-Wilk’s test). Descriptive statistics were presented as frequency, percentage, mean, median, and standard deviation values. Parameters determined to be normally distributed were compared using the One-way ANOVA test. Homogeneity of variances was evaluated with the Levene test. When there were differences between groups, the Tukey test was used for post-hoc comparisons. The nonparametric Mann-Whitney U test and the Kruskal-Wallis Test (post-hoc: Dunn-Bonferroni test) were used to compare groups that did not exhibit a normal distribution. Time-dependent changes in the parameters obtained from the groups were evaluated using repeated-measures analysis of variance. Statistical significance was defined as p < 0.05.

## 3. Results

This study was designed in five groups: control group (Group 1; n = 7), drowning in saltwater (Group 2; n = 7), immersion in saltwater (Group 3; n = 7), drowning in freshwater (Group 4; n = 7), and immersion in freshwater (Group 5; n = 7).

### 3.1. Serum

**Figure 2:**
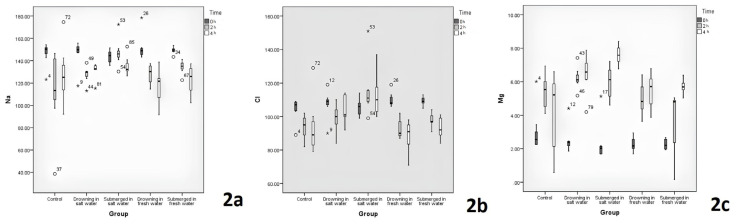
Variation of blood sodium, chlorine, and magnesium according to postmortem time.

The comparisons of serum Na, Cl, and Mg levels between the groups are displayed in Table 1. The highest Na level in the second-h was in saltwater immersion group. The saltwater immersion group’s second-h blood Na level was significantly higher than the saltwater drowning group (p = 0.015; post-hoc: p = 0.04). No remarkable difference was detected between the groups in blood Na level changes over time (p = 0.116) (Figure 2a). The highest Cl level in the second-h was in the saltwater immersion group. The saltwater immersion group’s second-h blood Cl level was significantly higher than the control and freshwater drowning group (p = 0.003, post-hoc: p = 0.02, p = 0.01, respectively). In the post-hoc test, there was no difference between the groups in fourth-h blood Cl (p = 0.002; post-hoc: p > 0.05). A significant difference was between the groups in blood Cl level changes over time (p < 0.001) (Figure 2b). The highest Mg level in the second-h was in the saltwater drowning group. The difference between the saltwater drowning and the freshwater immersion groups in the second-h blood Mg levels was statistically significant (p = 0.023, post-hoc: p = 0.02). The highest Mg level in the fourth-h was in the saltwater immersion group. The saltwater immersion group’s fourth-h blood Mg level was significantly higher than the control, freshwater drowning, and freshwater immersion groups (p = 0.001, post-hoc: p = 0.02, p = 0.03, p = 0.02, respectively). A remarkable discrepancy was between the groups in blood Mg level change over time (p < 0.001) (Figure 2c).

### 3.2. Vitreous humour

**Figure 3:**
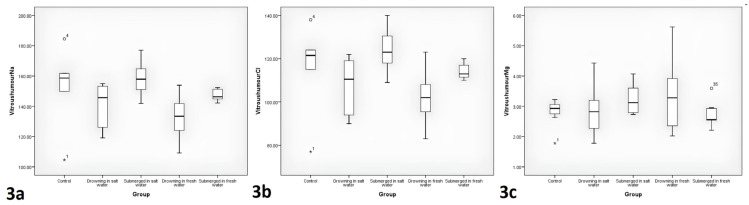
The comparisons of vitreous fluid Na, Cl, and Mg levels between the groups.

The comparisons of postmortem fourth-h vitreous fluid Na, Cl, and Mg levels between the groups are displayed in Table 1 and Figure 3. A difference was seen between the groups in fourth-h vitreous Na and Cl, but in the post-hoc test results of paired comparisons, it was not statistically significant (p > 0.05).

### 3.3. Aqueous humour

**Figure 4:**
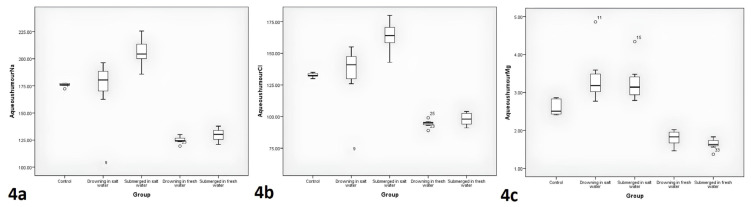
The comparisons of aqueous humour Na, Cl, and Mg levels between the groups.

The comparisons of the postmortem fourth-h aqueous humour Na, Cl, and Mg levels between the groups are displayed in Table 1 and Figure 4. The highest fourth-h aqueous humor Na level was in the saltwater immersion group, and the lowest was in the freshwater drowning group. The freshwater drowning and immersion groups’ fourth-h aqueous humor Na levels were significantly lower than the control and saltwater immersion groups (p < 0.001, post-hoc: p = 0.03, p = 0.02, respectively). Also, the saltwater immersion group’s fourth-h aqueous humor Na level was significantly higher than the control and the saltwater drowning groups (p < 0.001, post-hoc: p = 0.03, p = 0.04, respectively).

The highest fourth-h aqueous humor Cl level was in the saltwater immersion group, and the lowest was in the freshwater drowning group. The freshwater drowning and immersion groups’ fourth-h aqueous humor Cl levels were significantly lower than the control and saltwater immersion groups (p < 0.001, post-hoc: p = 0.03, p = 0.02, respectively). Also, the saltwater immersion group’s fourth-h aqueous humor Cl level was significantly higher than the control group and the saltwater drowning groups (p < 0.001, post-hoc: p = 0.03, p = 0.04, respectively).

The lowest fourth-h aqueous humor Mg levels were in the freshwater drowning and immersion groups. The freshwater drowning and immersion groups’ fourth-h aqueous humor Mg levels were significantly lower than the control group, saltwater drowning and immersion groups (p < 0.001, post-hoc: p = 0.03, p = 0.02, p = 0.02, respectively).

### 3.4. Corneal thickness and volume

**Figure 5:**
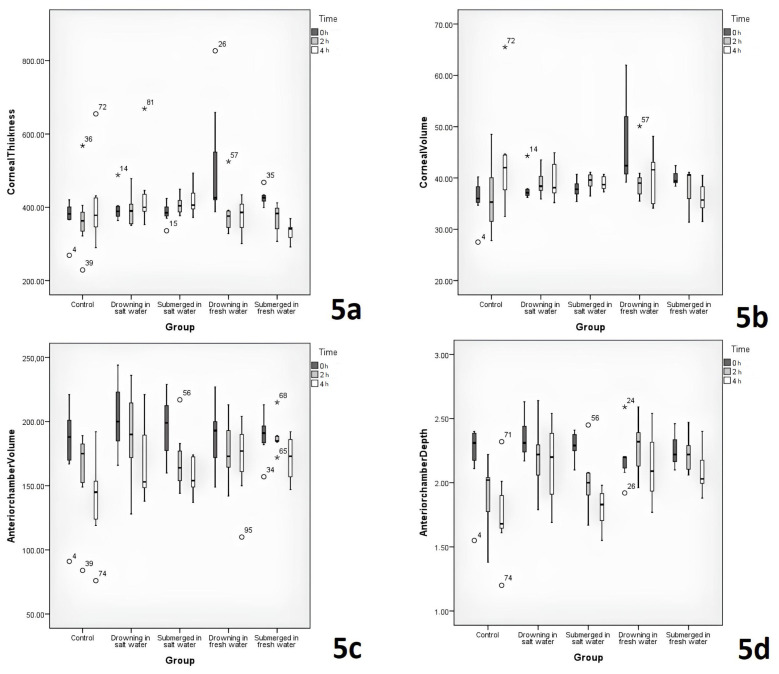
Variation of corneal thickness, corneal volume, anterior chamber volume, and anterior chamber depth according to postmortem time.

The comparisons of CT and CV levels between the groups are displayed in Table 2. The lowest fourth-h CT levels were in the freshwater immersion group. The freshwater immersion group’s CT level was significantly lower than the saltwater drowning and immersion groups (p = 0.019, post-hoc: p = 0.01). CT and CV changes according to time were significant differences between the groups (p = 0.003) (Figures 5a and 5b).

### 3.5. Anterior chamber volume (ACV) and anterior chamber depth (ACD)

The comparisons of ACV and ACD levels between the groups are displayed in Table 2. No remarkable difference was detected between the groups in the anterior chamber volume changes over time (p = 0.361) (Figure 5c). The second-h anterior chamber depth significantly differed between the freshwater drowning and the control groups (p = 0.026, post-hoc: p = 0.038). Although there was a difference in fourth-h ACD between the groups, the difference was not statistically significant in the post-hoc Tukey test (p > 0.05). There was a difference in fourth-h ACD between the groups, which was not statistically significant in the post-hoc Tukey test (p > 0.05). The ACD changes over time significantly differed between the groups (p = 0.001) (Figure 5d).

## 4. Discussion

Due to postmortem diffusion and dissociation, serum electrolyte changes are not completely reliable parameters [6]. Byard et al. [15] found a significant difference in left ventricular sodium levels between freshwater and saltwater drowning victims (mean 153 ± 14.4 for saltwater; 117 ± 14.2 for freshwater). Zhu et al. [8] separated 49 autopsy cases into freshwater drowning, saltwater drowning, and acute myocardial infarction, and analyzed serum Na, Cl, Mg, calcium (Ca), blood urea nitrogen (BUN), creatinine (Cr), pulmonary surfactant-associated protein A (SP-A) and cardiac troponin T (cTN-T). Left-right heart BUN levels were reported to be the most important marker in the determination of drowning (hemodilution) and the most important marker in the distinction between fresh-salt water was the left heart blood Mg level. Pérez-Carceles et al. [3] found that mean strontium (Sr), Cl, and Mg levels in both ventricles and serum, differences between left and right ventricles, and that left ventricular calcium and right ventricular sodium were significantly higher in drowning than in other causes of death. It was also suggested that serum values such as Mg and Cl may be helpful in the diagnosis of drowning in salt water, but blood Sr level provides the most significant data. In the current study, the saltwater immersion group’s second-h blood Na level was significantly higher than the saltwater drowning group (p = 0.015; post-hoc: p = 0.04) (Table 1). From these results it can be considered that blood Na levels may provide valuable information in the diagnosis of corpses pulled from the water in the early drowning period. Although there was a difference between the groups in the 2nd-h blood Cl values, this difference was due to the saltwater immersion group (p < 0.05) (Table 1). The data obtained in this study show that blood Cl values alone are not very useful in the drowning diagnosis. There was a significant difference between the blood Mg values of the groups at the 2nd and 4th h (p < 0.01) (Table 1). These differences were due to the freshwater immersion and saltwater immersion groups. Therefore, serum Mg levels cannot be used alone in the drowning diagnosis. In addition, the blood Cl and Mg level changes over time significantly differed between the groups (p < 0.001) (Figures 2b and 2c). Therefore, serum Cl and Mg levels vary depending on whether the corpse is in fresh or salt water, and may indicate whether the corpse was extracted from fresh or salt water.

Byard et al. [9] evaluated vitreous fluid samples from 2006 to 2009 to determine whether vitreous Na levels would be valuable in evaluating immersion-related deaths. In an autopsy series of 19 saltwater drowning and 16 freshwater drowning cases, vitreous fluid Na levels were between 145 and 184 mM in the saltwater drowning group and between 73 and 148 mM in the freshwater drowning group (p < 0.0001). The authors suggested that vitreous Na levels were an easily applicable test that may help investigate possible immersion deaths. In a study using bovine eyeballs, Anne et al. claimed that immersion did not cause any elevation in postmortem vitreous Na and Cl levels in bodies immersed in seawater for less than one h [16]. Cala et al. investigated the importance of postmortem vitreous Na and Cl levels to distinguish saltwater drowning from nondrowning immersion deaths from saltwater, and nonwater-exposure deaths [4]. It was reported that the combined postmortem vitreous Na and Cl levels were superior to the level of Na or Cl alone. It was argued that if the postmortem vitreous Na and Cl level was 284 mmol/L or higher and death conditions were compatible with drowning, a saltwater drowning diagnosis could be made. Garland et al. compared 24 cases of drowning in salt water and 96 control group cases who died due to natural causes and were removed from the water. The first-h postmortem vitreous Na and Cl levels were reported to be significantly higher in the saltwater drowning group than in the control group, and it was stated that postmortem vitreous Na and Cl levels of 259 mol/L and above in the first h could be a reliable adjunct test in saltwater drowning diagnosis [6]. The increase in first-h postmortem vitreous Na and Cl levels was said to be due to drowning, and the increase in the following hours was due to immersion. In the current study, there was not determined to be any significant difference in the posthoc test results of the paired comparisons in vitreous Na and Cl at the 4th h. The evidence of this study, in line with the literature, supports that vitreous Na and Cl levels cannot be used alone for a diagnosis of drowning in prolonged immersion.

In a study of 66 corpses retrieved from water, Tse et al. found that postmortem vitreous Mg levels were significantly higher in saltwater drowning >1 h than in saltwater drowning <1 h and nonimmersion deaths [17]. In the current study, no statistically significant difference was found between the groups in the vitreous fluid Mg levels taken at the 4th h (p > 0.05) (Table 1). These findings suggest that postmortem vitreous Mg, like Na and Cl, cannot be utilized alone to diagnose drowning after prolonged immersion.

Almost all of the studies in the literature have focused on vitreous fluid [4–17]. The findings obtained in this study, compatible with the literature, prove that vitreous fluid electrolytes are meaningless in long-term immersion in water. However, in this study, anterior chamber fluid (aqueous humour) was also taken together with vitreous fluid from the subjects at the postmortem 4th h. There is no previous study in the current literature that has studied aqueous humour. There were statistically significant differences between the groups in aqueous humour Na, Cl, and Mg levels taken at the 4th h (p < 0.001) (Table 1). These data demonstrate that aqueous humour Na, Cl, and Mg levels may be valuable in the diagnosis of drowning for identification corpses that have been in water for 4 h. It can be suggested therefore that aqueous humour Na and Cl levels may be valuable in drowning identification, especially in corpses taken from salt water (p < 0.001, post-hoc: p = 0.04). Also, low aqueous humour Na, Cl, and Mg levels may be essential as they indicate that the body remained in fresh water.

Previous studies have focused on serum, pericardial fluid, pleural fluid, vitreous fluid electrolyte changes, lung weight, and postmortem tomography findings for drowning identification [3–18]. In the current literature, CT, CV, ACV, and ACD have not been evaluated in drowning identification. The human cornea consists of five layers; three cellular (epithelium, stroma, endothelium) and two membranes (Bowman’s membrane, Descemet’s membrane). The endothelium (the most posterior layer of the cornea) is located between the anterior chamber and the cornea. The corneal endothelium is the cell layer that acts as a barrier by balancing the fluid in the cornea and acts as a pump for removing excess fluid from the cornea [19]. Based on this mechanism, it was aimed in this study, to determine the changes in the cornea and anterior chamber by performing saltwater drowning, saltwater immersion, freshwater drowning, and freshwater immersion experiments. Rabbit corneas also have epithelium, stroma, and endothelium similar to human corneas and are considered suitable for corneal experimental studies [20]. The CT, CV, ACV, and ACD of the rabbits in this study were evaluated with the Pentacam topography. The fourth-h corneal thicknesses significantly differed between the saltwater drowning group and freshwater immersion group and between the saltwater immersion group and the freshwater immersion group (p = 0.019, post-hoc: p = 0.01). Although the CV showed a significant difference between the groups at 0 h, there was no significant difference at the 2nd and 4th h (p > 0.05) (Table 2). However, there was a significant difference between the groups in the CT and CV changes according to time (p < 0.01) (Figures 5a and 5b). This may be due to the increase in thickness and volume of the cornea over time in saltwater cases, while it decreases over time in freshwater cases. CT and CV cannot be used alone in the diagnosis of drowning but may provide valuable information on the distinction between being in fresh water and salt water.

There was no significant difference in anterior chamber volume at 0, 2nd, and 4th h and changes over time (Table 2). However, there was a certain decrease in the change over time within all the groups (Figure 5c). There was a statistically significant difference in 2nd-h ACD between the freshwater drowning group and the control group (p = 0.026, post-hoc: p = 0.038) (Table 2). Although there was no significant difference between the groups in the change of ACD over time, there tended to be a decrease over time in all the groups (Figure 5d). ACV and ACD are not useful for drowning diagnosis. However, considering that these two data decrease regularly over time in all groups, it may be valuable to evaluate these two parameters in terms of time of death.

## 5. Limitations

Rabbits were used as experimental animals in this study, although they have different physical properties. Rabbit eyes are usually used as a surrogate in human ocular permeability studies. However, these experiments are instructive, as human and rabbit ocular permeability cannot be said to be the same. The findings should also be demonstrated on postmortem human subjects. Similar to any medical test, the findings of this study may also play only a helpful role in the diagnosis of drowning. Due to the complex nature of drowning, these findings are not diagnostic per se.

## 6. Conclusion

The results of this study demonstrated that CT, CV, ACV, and ACD are not of great value in drowning diagnosis and are affected by the fresh or salt water rather than drowning. This study supports previous studies in the literature showing that vitreous Na, Cl, and Mg levels will be ineffective in drowning after one h. However, Na, Cl, and Mg from the anterior chamber fluid were studied for the first time in this study. The data obtained suggest that anterior chamber fluid may provide valuable information in freshwater – saltwater drownings at the 4th h in corpses retrieved from the water. Anterior chamber fluid Na and Cl levels, especially in corpses removed from salt water, can be an easily used test that can help diagnose drowning. However, more extensive autopsy studies are required.

## Figures and Tables

**Table 1:** Comparison of Na, Cl, and Mg levels in serum, vitreous humour, and aqueous humour between groups according to time.

Serum (hours)	Group 1 (Control)		Group 2 (Drowning in salt water)		Group 3 (Submerged in salt water)		Group 4 (Drowning in fresh water)		Group 5 (Submerged in fresh water)		P*
	Mean	Median (Q1:Q3)	Mean	Median (Q1:Q3)	Mean	Median (Q1:Q3)	Mean	Median (Q1:Q3)	Mean	Median (Q1:Q3)	
**Na (0)**	146.24 ± 10.81	150.20 (142.60: 152.80)	146.12 ± 13.05	149.80 (147: 155)	143.57 ± 5.86	144.60 (137.4: 148.9)	151.95 ± 12.08	149 (144.6: 151.7)	149.11 ± 3.14	149.5 (148: 150.9)	0.411
**Na (2)**	113.15 ± 37.75	113.30 (97.50: 144)	127.57 ± 7.66	129.70 (124.2: 130.6)	147.51 ± 12.79	146 (140.9: 150.9)	127.84 ± 8.88	130.2 (120.2: 136.4)	134.21 ± 6.14	135.1 (110.7: 32.9)	0.015
**Na (4)**	127.34 ± 36.17	125.10 (109.30: 142.40)	130.76 ± 7.77	132.80 (127.7: 135.7)	135.45 ± 8.73	132.2 (130: 140.8)	116.27 ± 17.53	121.6 (92.8: 125.2)	122.61 ± 13.07	125.8 (110.7: 132.9)	0.173
**Cl (0)**	104.14 ± 7.08	107 (130: 109)	107.42 ± 8.73	109 (106: 111)	105.42 ± 5.15	106 (101: 108)	110.14 ± 4.52	108 (107: 113)	109.28 ± 2.75	109 (107: 112)	0.318
**Cl (2)**	93.57 ± 8.03	95 (83: 101)	99 ± 8.50	100 (94: 106)	115.71 ± 16.52	111 (108: 116)	92.85 ± 5.75	90 (88: 97)	98 ± 4.24	97 (96: 102)	0.003
**Cl (4)**	93.85 ± 17.1	89 (81: 100)	103.5 ± 8.45	101 (98: 113.25)	112.57 ± 12.99	110 (101: 120)	88.14 ± 10.18)	91 (77: 96)	93.28 ± 6.49	92 (88; 100)	0.002
**Mg (0)**	3.04 ± 1.37	2.54 (2.24; 3.44)	2.55 ± 0.84	2.38 (2.14; 2.46)	2.35 ± 1.24	2.03 (1.68; 2.18)	2.27 ± 0.42	2.17 (1.98; 2.55)	2.26 ± 0.32	2.2 (1.96; 2.57)	0.516
**Mg (2)**	5.37 ± 1.04	5.53 (4.27; 6.12)	6.21 ± 0.68	6.11 (5.93; 6.62)	5.94 ± 1.02	6.12 (4.73; 6.88)	5 ±1.01	4.82 (4.29; 6.23)	3.50 ± 2.19	4.80 (0.47; 4.97)	0.023
**Mg (4)**	4.05 ± 2.38	5.22 (2; 6.14)	6.44 ±1.18	6.58 (6.12; 7.39)	7.60 ± 0.61	7.58 (6.94; 8.26)	5.43 ± 1.13	5.71 (3.93; 6.34)	5.71 ± 0.42	5.69 (5.44; 5.95)	0.001
**Vitrous humour (hours)**	**Mean**	**Median (Q1;Q3)**	**Mean**	**Median (Q1;Q3)**	**Mean**	**Median (Q1;Q3)**	**Mean**	**Median (Q1;Q3)**	**Mean**	**Median (Q1;Q3)**	**P** *
**Na (4)**	152.98 ± 26.39	158.6 (138.57; 167.4)	140.8 ± 14.83	145.7 (124.35; 153.65)	158.34 ± 11.78	157.9 (149.8; 168.1)	132.6 ± 15.45	133.4 (117; 144.7)	147.55 ± 4.03	146.3 (144.3; 152.2)	0.027
**Cl (4)**	116.16 ± 20.66	121.5 (105.5; 127.5)	107.66 ± 13.06	110.5 (93; 19.75)	124.14 ± 10.41	123 (118; 133)	102.14 ± 13.14	102 (90; 102)	114.28 ± 3.72	113 (111; 117)	0.029
**Mg (4)**	2.79 ± 0.48	2.93 (2.63; 3.08)	2.85 ± 0.91	2.82 (1.87; 3.43)	3.24 ± 0.52	3.12 (2.77; 3.65)	3.35 ± 1.26	3.28 (2.26; 4.07)	2.78 ± 0.44	2.56 (2.54; 0.95)	0.585
**Aqueous humour (hours)**	**Mean**	**Median (Q1;Q3)**	**Mean**	**Median (Q1;Q3)**	**Mean**	**Median (Q1;Q3)**	**Mean**	**Median (Q1;Q3)**	**Mean**	**Median (Q1;Q3)**	**P** *
**Na (4)**	175.73 ± 1.87	176.05 (174.62; 177.25)	170.74 ± 32.71	180.4 (162.6; 195.4)	206.11 ± 12.97	204.4 (199.9; 217.7)	125.01 ± 3.52	124.4 (123.4; 128.7)	129.68 ± 5.93	130.2 (125.6; 133.9)	<0.001
**Cl (4)**	132.66 ± 1.75	132.5 (131.5; 134.25)	131.85 ± 28.32	141 (126; 154)	163.57 ± 12.03	164 (157; 174)	94.42 ± 3.04	95 (93; 96)	98 ± 5.16	98 (93; 103)	<0.001
**Mg (4)**	2.59 ± 0.20	2.50 (2.42; 2.83)	3.40 ± 0.69	3.18 (2.92; 3.59)	3.28 ± 0.52	3.14 (2.91; 3.48)	1.79 ± 0.20	1.83 (1.66; 1.96)	1.64 ± 0.14	1.63 (1.56; 1.74)	<0.001

* Kruskal-Wallis Test

**Table 2:** Comparison of corneal thickness, corneal volume, anterior chamber volume, and anterior chamber depth between groups according to time.

Corneal thickness (CorT) (hours)	Group 1 (Control)		Group 2 (Drowning in salt water)		Group 3 (Submerged in salt water)		Group 4 (Drowning in fresh water)		Group 5 (Submerged in fresh water)		P*
	Mean	Median (Q1;Q3)	Mean	Median (Q1;Q3)	Mean	Median (Q1;Q3)	Mean	Median (Q1;Q3)	Mean	Median (Q1;Q3)	
**CorT (0)**	372.42 ± 49.57	382 (366; 402)	399.57 ± 41.70	389 (370; 404)	385.71 ± 28.86	385 (370; 414)	511.85 ± 165.95	426 (419; 659)	427.42 ± 21.36	426 (415; 434)	0.005^1^
**CorT (2)**	371.85 ± 102.57	363 (322; 405)	392.28 ± 46.06	390 (354; 422)	406 ± 25.48	404 (382; 427)	385.42 ± 66.36	376 (334; 392)	368.57 ± 40.5	383 (322; 403)	0.331
**CorT (4)**	409.57 ± 119.49	378 (318; 431)	438.71 ± 105.78	400 (382; 446)	420 ± 42.08	406 (386; 458)	375.42 ± 47.05	386 (336; 410)	332.42 ± 25.29	341 (315; 348)	0.019
**Corneal volume (CorV) (hours)**	**Mean**	**Median (Q1;Q3)**	**Mean**	**Median (Q1;Q3)**	**Mean**	**Median (Q1;Q3)**	**Mean**	**Median (Q1;Q3)**	**Mean**	**Median (Q1;Q3)**	**P** *
**CorV(0)**	35.82 ± 4.14	36 (34.7; 39.1)	38.05 ± 2.82	37.1 (36.4; 37.9)	37.91 ± 1.74	37.8 (36.5; 39)	47.01 ± 9.68	42.4 (40.2; 60)	40.01 ± 1.42	39.4 (39; 41.3)	0.001
**CorV (2)**	36.4 ± 7.04	35.3 (30.4; 40.4)	39.08 ± 2.53	38.4 (36.9; 40.6)	39.32 ± 1.78	39.6 (37.3; 41.1)	39.78 ± 4.94	39 (35.7; 40.9)	38.04 ± 4.07	40.6 (33; 40.6)	0.688
**CorV (4)**	43.47 ± 10.61	42 (37.6; 44.7)	39.67 ± 3.80	38.1 (36.3; 44.2)	39.01 ± 1.37	38.7 (37.8; 40.5)	39.98 ± 5.38	41.6 (34.2; 43.8)	36.1 ± 3.32	35.7 (33.1; 40.1)	0.273
**Anterior chamber volume (ACV) (hours)**	**Mean**	**Median (Q1;Q3)**	**Mean**	**Median (Q1;Q3)**	**Mean**	**Median (Q1;Q3)**	**Mean**	**Median (Q1;Q3)**	**Mean**	**Median (Q1;Q3)**	**P** 1
**ACV (0)**	177.42 ± 42.83	188 (167; 213)	203.71 ± 28.61	200 (179; 236)	195.42 ± 25.38	199 (167; 216)	185.57 ± 25.93	193 (165; 206)	188.71 ± 17.28	191 (182; 199)	0.546
**ACV (2)**	159.71 ± 36.58	175 (149; 187)	189.57 ± 36.77	190 (163; 223)	169.57 ± 24.83	164 (147; 183)	177.57 ± 25.27	173 (161; 208)	188.28 ± 13.03	185 (184; 189)	0.271
**ACV (4)**	138.28 ± 35.89	145 (119; 154)	169 ± 31.38	153 (148; 202)	158.28 ± 14.79	154 (146; 173)	170.42 ± 32.16	177 (150; 200)	171.14 ± 17.71	173 (154; 187)	0.155
**Anterior chamber debth (ACD) (hours)**	**Mean**	**Median (Q1;Q3)**	**Mean**	**Median (Q1;Q3)**	**Mean**	**Median (Q1;Q3)**	**Mean**	**Median (Q1;Q3)**	**Mean**	**Median (Q1;Q3)**	**P** 1
**ACD (0)**	2.19 ± 0.30	2.31 (2.11; 2.39)	2.35 ± 0.16	2.31 (2.2; 2.49)	2.29 ± 0.10	2.29 (2.22; 2.38)	2.19 ± 0.20	2.2 (2.08; 2.2)	2.25 ± 0.12	2.22 (2.14; 2.35)	0.502
**ACD (2)**	1.89 ± 0.28	2.02 (1.66; 2.04)	2.19 ± 0.26	2.22 (2.01; 2.36)	2.01 ± 0.23	2 (1.86; 2.08)	2.27 ± 0.21	2.32 (2.12; 2.44)	2.22 ± 0.14	2.22 (2.07; 2.32)	0.026
**ACD (4)**	1.75 ± 0.34	1.68 (1.61; 2.01)	2.14 ± 0.32	2.20 (1.86; 2.54)	1.80 ± 0.15	1.83 (1.65; 1.93)	2.12 ± 0.27	2.09 (1.84; 2.35)	2.09 ± 0.18	2.03 (1.99; 2.28)	0.019

* Kruskal-Wallis Test

1 One-Way ANOVA
